# Protein folding and glycosylation process are influenced by mild hypothermia in batch culture and by specific growth rate in continuous cultures of CHO cells producing rht-PA

**DOI:** 10.1186/1753-6561-7-S6-P108

**Published:** 2013-12-04

**Authors:** Mauricio Vergara, Silvana Becerra, Julio Berrios, Juan Reyes, Cristian Acevedo, Ramon Gonzalez, Nelson Osses, Claudia Altamirano

**Affiliations:** 1 Escuela Ingeniería Bioquímica, Pontificia Universidad Católica de Valparaíso, Valparaíso, 2362806, Chile; 2 Centro Regional En Alimentos Saludables (CREAS), Valparaíso, 2340025, Chile; 3 Instituto Química, Pontificia Universidad Católica de Valparaíso, Valparaíso, 2340025, Chile; 4 Centro de Biotecnología, Universidad Técnica Federico Santa María, Valparaíso, 2390123, Chile; 5 Department of Chemical and Biomolecular engineering, RICE University, Houston, 77055, USA

## Background

CHO cells are the primary host for the production of different biopharmaceuticals, including recombinant proteins, monoclonal antibodies, vaccines, etc. Primarily due to their ability to perform properly folding and glycosylation processes required for these proteins acquire adequate biological functionality.

However, culturing of these cells in the bioreactor still presents a number of disadvantages, among which can be mention: nutrient depletion, toxic byproducts accumulation, limited oxygen transfer, etc. These issues limit the cell growth and early onset of programmed cell death, which restricts the longevity of cultures and jointly specific productivity of recombinant protein.

To overcome these limitations, different approaches have been made to maximize the productivity of these cultures. One of these approaches, that has gained importance during the last 20 years is the use of mild hypothermic temperatures, within a range of 33°C to 30°C. This strategy has been demonstrated to reduce the rate of growth and metabolism of cells but in turn increases the longevity of cultures and increase in specific productivity of a wide range of recombinant proteins in batch cultures [1,2].

One possible cause involved in the increase of specific productivity of recombinant proteins, is the increase in folding capacity and expression of chaperones from endoplasmic reticulum [3,4]. However, the intracellular mechanisms underlying the effect of temperature on the stages of post-translational protein synthesis are still poorly understood.

In this regard, the study of endoplasmic reticulum processes (folding, assembly and glycosylation of proteins, and degradation of misfolded proteins through ERAD pathway) has reached a high interest in recent years [4,5]. Reports show that the expression of several proteins associated with the various processes that take place in the ER, are affected under conditions of mild hypothermia. However, this phenomenon has not been analyzed from a process perspective.

Thus, this study investigated the effect of mild hypothermic temperatures (33°C) on the process of protein folding of rht-PA expressed in CHO cells. For this, inhibitors of protein translation, glycosylation and endoplasmic reticulum associated degradation pathways (ERAD I: via the ubiquitin/proteasome and ERAD II: autophagosome/Lysosome) were used. Two experimental approaches were evaluated: batch culture and continuous culture.

## Materials and methods

### Batch Culture

CHO cells were cultured in HyClone SFM4CHO medium with out glucose, supplemented with 20 mM glucose, at 95% relative humidity in an atmosphere of 5% CO2, at temperatures of 37°C or 33°C. The inhibitors used to block processes in the endoplasmic reticulum were: cycloheximide (Sigma, C4859)-protein translation; tunicamycin (Sigma, T7765)-N-glycosylation of proteins, MG132 (Merck, 474790)-ERAD I pathway; Pepstatin A (Merck, 516485), Leupeptin (Merck, 108976) and E64d (Sigma, E8640)-ERAD II pathway.

### Continuous culture

The bioreactor was inoculated and operated in batch-mode during 48 h and it was then supplied with sterile feed throughout the period of operation. A series of four experiments was performed, in duplicate, at 37°C or 33°C, keeping D, at 0.014 and 0.012 h^-1^. Cultures were considered to reach steady-state (SS) when, after at least four residence times, both, the number of viable cells and lactate concentration, were constant in two consecutive samples.

Cell growth was measured by counting cells by trypan blue method; consumption and production of metabolites were measured by biochemical analyzer (YSI 2700); protein rht-PA was measured by ELISA (Trinilize tPA antigen) and enzymatic activity of the protein was measured by amidolytic assay (S-2288 peptide, Chromogenix Italy). The results were analyzed by the mathematical technique of PCA (Principal Component Analysis).

## Results

The results of the batch cultures may indicate that the process of protein folding is sensitive to mild hypothermia. Inhibition of glycosylation process and ERAD pathways (ERAD I or II), under conditions of low temperature, promotes the accumulation of intracellular deglycosylated rht-PA as shown in Table 1. This response may indicate that the protein folding process is attenuated under conditions of mild hypothermia, promoting unfolded protein degradation by both ERAD pathways in CHO cells.

**Table 1:** Intracellular rht-PA content (% of control) on CHO cells by inhibition of translation and glycosylation prosesses and ERAD I and II pathways at 37°C and 33°C.

				Dilution rate (h^-1^)			
				0.014		0.012	
	Temperature			Temperature		Temperature	
Batch Cultures	37°C	33°C	Continuous Cultures	37°C	33°C	37°C	33°C
CC	100^1^	100^2^	SS	100^3^	100^4^	100^5^	100^6^
TM*	107	140	CHX/ERAD I**	120	117	185	139
TM/ERAD I*	87	201	CHX/ERAD II**	107	115	242	150
TM/ERAD II*	79	176					

CC: Control Culture; TM: Culture inhibited glycosylation; TM/ERAD I or II: Culture inhibited glycosylation and ERAD I or II; SS: Steady State; CHX/ERAD I or II: Culture inhibited translation and ERAD I or II; *Values at 24 hours after perturbation with inhibitors respect to CC value at 0 h. **At 48 hours after perturbation with inhibitors respect to value at SS.

^1^Concentration (8,8 ng/10^6 ^cells).

^2^Concentration (8,6 ng/10^6 ^cells).

^3^Concentration (7,9 ng/10^6 ^cells).

^4^Concentration (6,5 ng/10^6 ^cells).

^5^Concentration (4,2 ng/10^6 ^cells).

^6^Concentration (6,1 ng/10^6 ^cells).

Recent reports [6,7] show that the effect of mild hypothermia condition in batch culture is associated predominant with a decrease on specific cell growth rate rather a decrease on culture temperature. To evaluate this fact, we carried out continuous cultures at different dilution rates.

These results show that the degradation of the protein would be more related to the decrease in specific growth rate than the temperature decrease. Also show that the temperature decrease would promote an increase in protein folding capacity of the endoplasmic reticulum. This fact is clearly observed at low specific growth rate (Table 1).

**Figure 1:**
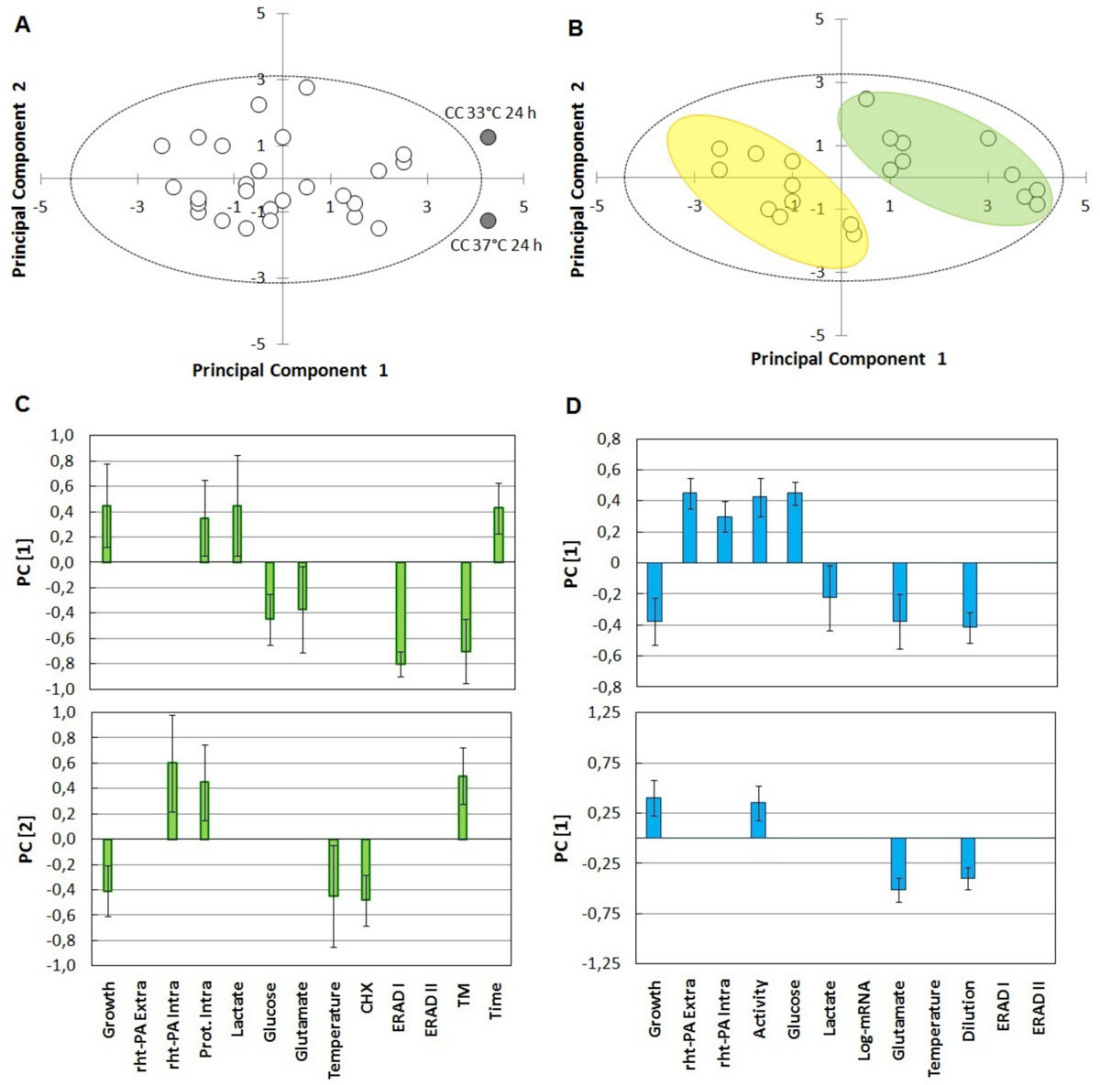
**First principal plane and Loads of first and second principal component of Batch and Continuous cultures**. **A: **First principal plane of Batch culture **B: **First principal plane of continuous culture; **C: **Loads of first and second principal component of Batch cultures. **D: **Loads of first and second principal component of Continuous cultures.

The cell behavior was evaluated using the technique of principal component analysis (PCA) in both, batch and continuous culture Figure 1.

The first principal plane (PC1 axis and PC2 axis) of batch cultures (Figure 1A) shows that there are only two values whose behavior is significantly away from the origin (P < 0,05). These correspond to the behavior of the tested batch cultures at 24 h at 37°C and 33°C, respectively. This indicates the great influence of culture temperature on cell behavior. The first principal plane of continuous culture (Figure 1B) shows the behavior of cells organized into two major groups, which are correlated with both dilution rates tested.

PC1 loads of batch cultures (Figure 1C) suggest that low temperature reduces the ability of the protein folding; this would explain the accumulation of intracellular deglycosylated rht-PA. However, loads of PC1 from continuous cultures (Figure 1D) shows that increasing of intracellular rht-PA content is associated with the reduction in the rate of dilution and is not associated with a lower temperature.

## Conclusions

Experimental approach of continuous culture revealed that reduction on specific growth rate is associated to an increase ERAD activity on rht-PA while the temperature reduction may have a positive effect on protein folding. Moreover, PCA analysis indicated that specific growth rate is also responsible for general behavior exposed by CHO cells.

## References

[B1] Yoon SK Song JY Lee GM Effect of low culture temperature on specific productivity, transcription level, and heterogeneity of erythropoietin in Chinese hamster ovary cells Biotechnol Bioeng 2003 7 289 298 12599255 10.1002/bit.10566

[B2] Bollati-Fogolín M Forno G Nimtz M Conradt HS Etcheverrigaray M Kratje R Temperature reduction in cultures of hGM-CSF-expressing CHO cells: effect on productivity and product quality Biotechnology Progress 2005 7 17 21 15903236 10.1021/bp049825t

[B3] Baik JY Lee MS An SR Yoon SK Joo EJ Kim YH Park HW Lee GM Initial Transcriptome and Proteome Analyses of Low Culture Temperature-Induced Expression in CHO Cells Producing Erythropoietin Biotechnol Bioeng 2006 7 361 371 16187333 10.1002/bit.20717

[B4] Masterton RJ Roobol A Al-Fageeh M Carden M Smales CM Post-Translational Events of a Model Reporter Protein Proceed With Higher Fidelity and Accuracy Upon Mild Hypothermic Culturing of Chinese Hamster Ovary Cells Biotechnol Bioeng 2010 7 215 220 19739092 10.1002/bit.22533

[B5] Gomez N Subramanian J Ouyang J Nguyen M Hutchinson M Sharma VK Lin AA Yuk IH Culture Temperature Modulates Aggregation of Recombinant Antibody in CHO Cells Biotechnol Bioeng 2012 7 125 136 21965146 10.1002/bit.23288

[B6] Becerra S Berrios J Osses N Altamirano C Exploring the effect of mild hypothermia on CHO cell productivity Biochem Eng J 2012 7 1 8

[B7] Vergara M Becerra S Berrios J Osses N Reyes J Rodríguez-Moyá M Gonzalez R Altamirano C Differential effect of culture temperature and specific growth rate on CHO cell behavior in continuous culture Bioch Eng J 2013 submitted 10.1371/journal.pone.0093865 PMC3974816 24699760

